# New Insight into Mixing Fluoride and Chloride in Bioactive Silicate Glasses

**DOI:** 10.1038/s41598-018-19544-2

**Published:** 2018-01-22

**Authors:** Xiaojing Chen, Xiaohui Chen, Alfonso Pedone, David Apperley, Robert G. Hill, Natalia Karpukhina

**Affiliations:** 1 Xiangya Stomatological Hospital & School of Stomatology, Central South University, Changsha, Hunan 410078 P.R. China; 2 Dental Physical Sciences, Institute of Dentistry, Queen Mary University of London, Mile End Road, London, E1 4NS United Kingdom; 3 Division of Dentistry, School of Medical Sciences, University of Manchester, Manchester, M13 9PL United Kingdom; 4 Dipartimento di Scienze Chimiche e Geologiche, Università di Modena e Reggio Emilia, Via G. Campi 103, 41125 Modena, Italy; 5 Department of Chemistry, Durham University, South Road, Durham, DH1 3LE United Kingdom

## Abstract

Adding fluoride into bioactive glasses leads to fluorapatite formation and a decrease in glass transition temperature. Recently, chloride has been introduced into glasses as an alternative to fluoride. The presence of the large chloride ion lowers glass crystallisation tendency and increases glass molar volume, which effectively facilitates glass degradation and bone-bonding apatite-like layer formation. However, there is no information regarding the effect of mixing fluoride and chloride on the glass structure and properties. This study aims to synthesize mixed fluoride and chloride containing bioactive glasses; investigate the structural role of fluoride and chloride and their effects on glass properties. The chloride content measurements reveal that 77–90% of chloride was retained in these Q^2^ type glasses. Glass transition temperature reduced markedly with an increase in CaX_2_ (X = F + Cl) content, while the glass molar volume increased. ^29^Si MAS-NMR results show that the incorporation of mixed fluoride and chloride did not cause significant change in the polymerization of the silicate network and no detectable concentration of Si-F/Cl bands were present. This agrees with ^19^F NMR spectra showing that F existed as F-Ca(n) species.

## Introduction

Bioactive glasses (BGs) are well known for their bone regenerative properties^1,2^. When immersed in physiological solutions, they degrade, release ions and form an integrated bond with living bone via the formation of an apatite layer on the surface. They are of interest for applications in soft tissue repair due to their ability of promoting angiogenesis (formation of blood vessels)^3–5^; bone grafting^6^, toothpastes^7^ and as dental air abrasives^8^. Certain glass properties are desired for specific applications. For example, it is thought that highly degradable glasses are favourable for resorbable bone grafting materials^9^, while BGs that release fluoride and form fluorapatite (FAP) are preferred for re-mineralizing toothpastes and the BGs with controllable hardness are particularly attractive for selective cutting of dental tissues. It is believed that glass composition and especially its structure defines its properties^10^. Therefore, the knowledge of structure-property relationship is required in order to tailor the glass properties to the preferred applications.

In recent years, there is an increase amount of interest in halogen-containing oxide glass systems, due to their unique mechanical, optical, electrical and medical properties. Fluoride containing BGs were first investigated by Hench and Spillman^11^ who substituted CaF_2_ for CaO or Na_2_O. It is noted that this approach results in an increase in the polymerization of the silicate tetrahedra^12,13^ and the network connectivity, which substantially reduces the glass bioactivity^14^. In contrast to replacing oxides with CaF_2_, adding calcium fluoride into BGs leads to no change in the glass Q silicate network structure^15,16^. The incorporation of fluoride into the BGs reduces glass firing temperature, glass transition temperature (T_g_) and probably glass hardness^17,18^. More recently, Sriranganathan *et al*.^18^ demonstrated that the presence of fluoride promotes and speeds up FAP formation upon immersion in buffer solution, this effect is more pronounced in Sr containing glasses. Fluorapatite is well-documented to be less soluble in an acidic condition than hydroxyapatite^19–22^. However, high fluoride content (≥9.3 mol%) results in an uncontrollable *fluorite* (CaF_2_) crystallisation^17^. The insoluble CaF_2_ will inhibit the delivery of fluoride ions from glass and also reduce glass bioactivity^23^.

Chloride volatilization is a severe problem in silicate glasses and results in very few studies on oxychloride silicate glasses and very rare practical applications, though chloride has been used as a refining aid in glass melting^24^. Recently, chloride has been introduced to the sodium-free bioactive silicate glasses as an alternative to fluoride by Chen *et al*.^9,25,26^. A considerable amount of chloride (up to 16.7 mol%) has been retained in the BGs. It is found that adding chloride leads to a reduced T_g_ in a similar manner to fluoride and a significant expansion of glass molar volume, which is beneficial to fast glass degradation and rapid apatite-like phase formation *in vitro*. Moreover, the crystallisation tendency of the chloride containing glasses was much lower than the equivalent fluoride containing glasses. All the studied chloride containing glasses were largely amorphous, this shows a clear contrast to the equivalent fluoride glasses, which crystallised to fluorapatite, cuspidine and fluorite when the fluoride content were higher than 9.3 mol%^17^.

It is known that the chloride ion is larger than the hydroxyl ion, which is larger than the fluoride ion (R_ion_ (Cl^−^):R_ion_ (OH^−^):R_ion_ (F^−^) = 1.67:1.26:1.19 (Å)). As a result, chlorapatite is more soluble than hydroxyapatite, which is more soluble than fluorapatite^25^. Additionally, unlike the formation of fluorapatite in fluoride containing glasses, a hydroxyapatite-like phase was the phase formed in the chloride containing glasses upon immersion^9^. This is favorable for resorbable bone substitutes but less attractive for the toothpastes for re-mineralization and caries protection. BGs with the presence of fluoride and chloride are of special interest, since they are potentially able to achieve the benefits from both. However, to the best of our knowledge, mixed fluoride and chloride containing BGs have not been yet synthesized and investigated in the literature and the structure of fluorine and, especially chlorine in silicate glasses remains unclear.

Brauer *et al*.^27^ has investigated the structural role of fluoride by using ^19^F MAS-NMR, which showed that fluoride complexes the modifier cations (Ca^2+^ and Na^+^) rather than forming Si-F bonds. Chloride is expected to have similar effects on the glass structure and properties to fluoride. ^35^Cl MAS-NMR is potentially a promising technique to provide a direct view of the chlorine environment on an atomic-scale. However, ^35^Cl MAS-NMR is not as straightforward as ^19^F MAS-NMR. ^35^Cl is a spin 3/2 low γ nuclide with a large quadrupole moment and a relatively low resonance frequency^28^. In addition chloride often exhibits a low solubility in silicate melts^29^. As a result of these problems, ^35^Cl MAS-NMR signals can be severely broadened and indiscernible from background, and require very high magnetic fields and the use of fast spinning frequencies. Therefore, to date, only a few direct studies on the atomic environment of chloride have been reported^28,30^. In general, these studies indicate that Cl is coordinated primarily by alkali or alkaline earth cations.

In this paper, the possibility of incorporating both fluoride and chloride into glass GPFCl0.0 (38.1% SiO_2_, 6.3% P_2_O_5_, 55.5% CaO, in mol%) and their effects on the glass structure and properties were investigated and compared with only fluoride or chloride containing BGs (GPF and GPCl series) studied previously^9,23^. Various techniques, including Differential Scanning Calorimetry, X-ray Diffraction, Magic Angle Spinning-Nuclear Magnetic Resonance (^29^Si, ^31^P and ^19^F MAS-NMR) and Helium Pycnometer were employed to understand the glass structure and properties. To the best of our knowledge, this is the first study on mixed chloride/fluoride containing BGs.

## Materials and Methods

### Glass synthesis

Novel mixed fluoride and chloride containing BGs in the system of SiO_2_-P_2_O_5_-CaO-CaF_2_/CaCl_2_ were prepared by the melt-quench route. Instead of replacing CaO by CaX_2_ (X = F + Cl), a varied amount of CaX_2_, which is consist of 50% CaF_2_ and 50% CaCl_2_·2H_2_O, since CaCl_2_ picks up water easily, was added to a calcium halide free composition (GPFCl0.0)^9^, whilst other components (SiO_2_, P_2_O_5_ and CaO) were reduced proportionally. All the glasses were designed to have a constant network connectivity (NC) value of 2.08. Glass compositions are reported in Table 1. A 200 g batch size was made. Glass reagents including analytical grade SiO_2_ (Prince Minerals Ltd., Stoke-on-Trent, UK), CaCO_3_, P_2_O_5_, CaF_2_ and CaCl_2_.2H_2_O (all Sigma-Aldrich) were melted at high temperatures in a Pt/10Rh crucible for 1 hour in an electrical furnace (EHF 17/3 Lenton, Hope Valley, UK). The melted glass was rapidly water quenched. The collected glass frits were dried, Gy-Ro milled (Glen Creston, London, UK) and sieved through a 45 μm mesh analytical sieve (Endecotts Ltd, London, England). The compositions of the individual chloride and fluoride series, which have been reported previously^9,23^, are presented in ESI (Table S1) for comparison purpose.

**Table 1:** Compositions of the Experimental Glasses in Mol%. For each glass, the first row is the nominal composition as-designed and the second row is composition re-calculated based on the chloride component analysis and assumed chlorine losses as CaCl_2_.

Glass code	SiO_2_	CaO	P_2_O_5_	CaF_2_	CaCl_2_	Total CaX_2_	T_firing_ (°C)	NC
GPFCl0.0	38.1	55.5	6.3	0.0	0.0	0.0	1550	2.08
	38.1	55.6	6.3	0.0	0.0	0.0		
GPFCl2.6	37.1	54.1	6.1	1.5	1.1	2.6	1520	
	37.2	54.2	6.1	1.5	0.9	2.4		
GPFCl4.0	36.6	53.4	6.0	2.3	1.7	4.0	1500	
	36.8	53.5	6.1	2.3	1.4	3.7		
GPFCl5.3	36.2	52.6	5.9	3.0	2.3	5.3	1500	
	36.3	52.8	6.0	3.0	1.9	4.9		
GPFCl8.3	35	51.0	5.8	4.7	3.6	8.3	1500	
	35.1	51.2	5.8	4.7	3.2	7.9		
GPFCl12.1	33.5	48.8	5.6	6.9	5.2	12.1	1500	
	33.8	49.3	5.6	7.0	4.3	11.3		
GPFCl16.0	32.1	46.7	5.3	9.1	6.9	16.0	1500	
	32.4	47.1	5.4	9.2	6.0	15.2		
GPFCl23.1	29.3	42.7	4.9	13.2	9.9	23.1	1500	
	29.9	43.6	5.0	13.4	8.1	21.5		

### Compositional analysis

The chloride content in the initial mixed glasses was quantified using a chloride ion selective electrode (ELIT Cl- 2844, NICO 2000 UK), according to the method described by Chen *et al*.^9^. The actual glass compositions were re-calculated based on the chloride component analysis and summarized in Table 1.

### Glass characterisation

Glass thermal properties were evaluated by using a Stanton Redcroft DSC 1500 (Rheometric Scientific, Epsom, UK). A 50 mg of glass frit was heated under Nitrogen (60 ml/min^−1^) from 25 °С to 1100 °С at a rate of 20 °С/min against an alumina reference in a Pt crucible. T_g_ value was extracted from the DSC traces with an accuracy of ±5 °С.

An X’Pert Pro X-ray diffractometer (PANalytical, Eindhoven, The Netherlands) was used to investigate the amorphous status of studied glasses and their crystalline phases. The powder samples were scanned from 5 to 70° 2θ with an interval of 0.0334° and a step time of 200.03 sec.

^29^Si, ^31^P and ^19^F Magic Angle Spinning-Nuclear Magnetic Resonance (MAS-NMR) were employed to characterize the glass structure. ^29^Si MAS-NMR spectra were acquired on a VNMRS 400 (9.4 T) spectrometer, while the ^31^P and ^19^F MAS-NMR spectra were collected on a AVANCE 600 MHz (14.1 T) Bruker NMR spectrometer. A resonance frequency of 79.4 MHz was used for ^29^Si MAS-NMR. Glass powder was packed in a 6 mm zirconia rotor and spun at 6 kHz with a relaxation time of 300 s delay for 432 scans and a pulse duration of 4.0 μs. The isotopic chemical shift was referenced using tetramethylsilane Si(CH_3_)_4_) solution. ^31^P MAS-NMR experiments were performed at a Larmor frequency of 242.9 MHz with spinning condition of 8 kHz in a 4 mm zirconia rotor. A recycle delay of 60 s and a scan number of 16 were used. The chemical shift was referenced to 0 ppm frequency of the corresponding signal of 85% H_3_PO_4_. ^19^F MAS-NMR spectra were conducted at the Larmor frequency of 564.7 MHz using a double resonance Bruker probe with a low fluorine background and tunable to the ^19^F NMR frequency under spinning conditions of 18 kHz or 21 kHz using a 2.5 mm rotor. After the 8 preliminary dummy scans, either 32 or 64 scans were acquired with 30 s recycling delay. The chemical shift of ^19^F was referenced to a −120 ppm frequency of the signal from the 1 M aqueous solution of NaF relative to the primary standard, CFCl_3_.

Glass density was determined by Helium Pycnometry (AccuPyc 1330–1000, Micromeritics, GmbH, Aachen, Germany) with a pressure at 1.6 bar. Approximately 2 g of fine glass powder (<45 µm) was used to carry out the measurement. The presented density values are the mean of ten measurements performed during the experiment.

The glass molar volume (*V*_*m*_) of the glasses was calculated by using the equation^31^:

1 $${V}_{m}=M/D$$

Where *M* is the relative molecular mass of glass and *D* is the experimental density.

### Data availability

The datasets generated during and/or analysed during the current study are available from the corresponding author on reasonable request.

## Results and Discussion

### Compositional analysis

**Figure 1:**
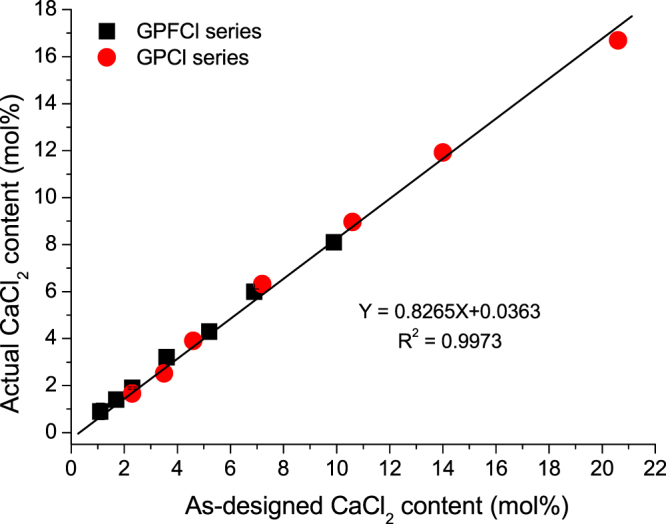
The percentage of the retained chloride in the initial glasses plotted against the as-designed CaCl_2_ content for GPFCl and GPCl glass series. Note where error bars are not shown they are smaller than the data point.

The actual chloride content in the mixed GPFCl glasses is plotted against the as-designed CaCl_2_ content in Fig. 1. The data fits well to a linear regression. The trend is similar to the retention of chloride in the glasses containing chloride alone (GPCl series) reported earlier which are also added to the Fig. 1^9^. The linear fit and equation presented in the figure take into account overall data in both series. It is seen that a large fraction of chloride is retained in both glass series. The slope of the linear fit gives chloride retention of about 82.5%. As shown earlier in the GPCl series, chloride is likely to evaporate mainly as CaCl_2_ rather than HCl. A similar scenario is expected in the GPFCl series. Thus, based on this assumption and taking into account the results of chloride quantitative analysis, the actual glass compositions for the GPFCl series were recalculated and presented in Table 1.

### Glass transition temperature

**Figure 2:**
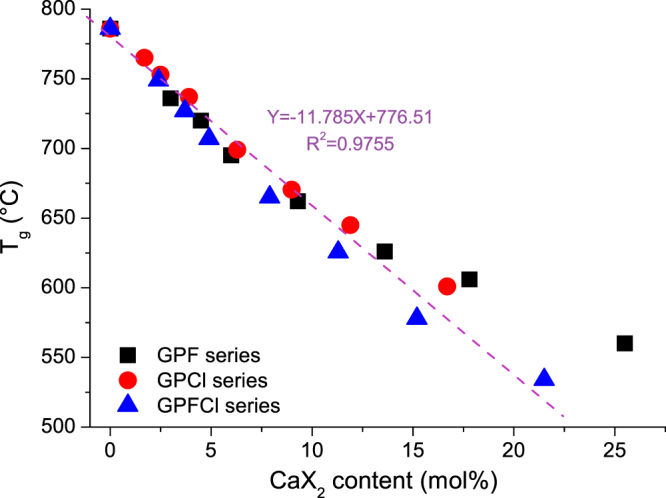
Glass transition temperature of the frit profiled as a function of the actual mixed CaX_2_ (X = F + Cl) content. A linear relationship (Y = −11.785x + 776.51, R^2^ = 0.9755) between T_g_ and CaX_2_ content was shown in all three glass series with the exception of glass compositions GPF17.8 and GPF25.5, which are largely crystallised. The data for GPF and GPCl series previously reported^9,17^ are shown here for a comparison purpose.

The glass transition temperature of frit for GPFCl series is plotted as a function of the actual mixed CaF_2_ and CaCl_2_ content and compared with GPF and GPCl glass series in Fig. 2. It is clear that T_g_ decreases linearly with an increase in CaX_2_ content in all glass series. This strong linear relationship points out a similar retention/loss percentage of the halide component in all three series. The last two compositions of the fluoride series GPF25.5 and GPF17.8 deviate from the linear trend. This is believed due to a substantial crystallisation that occurs in these compositions discussed previously^17^.

### Glass density

**Figure 3:**
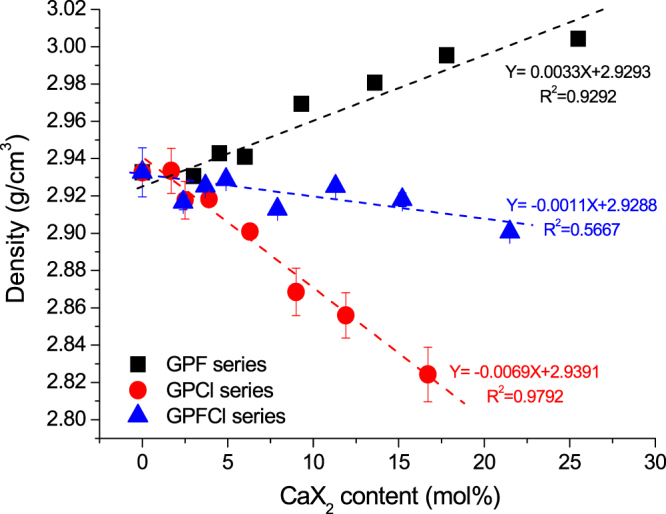
Glass density profiled as a function of CaX_2_ (X = F/Cl/F + Cl) content. Linear relationships (Y = 0.0033x + 2.9293, R^2^ = 0.9292 and Y = −0.0069x + 2.9391, R^2^ = 0.9792) between density and CaX_2_ content are shown in GPF and GPCl glass series. Note that the line crossing the density values for the GPFCl series is applied to illustrate the general trend of the data.

Glass density values of the GPFCl glass series are plotted against CaX_2_ content and compared with the density values of the GPF and GPCl series, which have been reported previously^9,23^ in Fig. 3. The figures reveal three different trends becoming clearer for high halide content. The experimental density values are relatively close to each other for all three series up to about 5 mol% of CaX_2_ content. A clear increase in density is visible with increasing CaF_2_ content for the GPF glass series, similar trend was found in SiO_2_-P_2_O_5_-CaO-Na_2_O-CaF_2_ glass system by Brauer *et al*.^31^. Conversely, the density of GPCl glass series decreases with an increase in CaCl_2_ content. The density values of GPFCl glass series fall in between the equivalent of GPF glass series and GPCl glass series. The values fluctuate between 2.9 and 2.93 g/cm^3^ and display a relatively weak dependence on CaX_2_ content, though strong linear relationships were found for the other two individual calcium halide containing glass series (GPF and GPCl series).

### Glass molar volume

**Figure 4:**
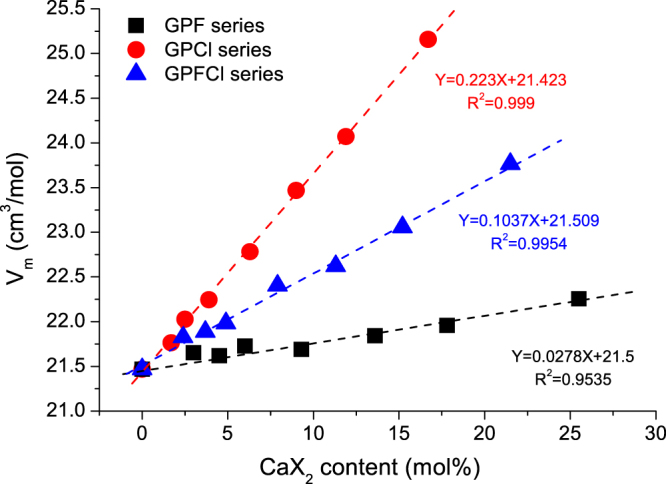
Glass molar volume profiled as a function of CaX_2_ (X = F/Cl/F + Cl) content. Linear relationships (Y = 0.223 x  + 2.21.423, R^2^ = 0.999, Y = 0.1037 x  + 21.509, R^2^ = 0.9954 and Y = 0.00278 x  + 21.5, R^2^ = 0.9535) between density and CaX_2_ content are shown as dotted lines in GPF, GPCl and GPFCl glass series.

Figure 4 shows the glass molar volume of the three series of glasses plotted as a function of CaX_2_ content. It is interesting to find that *V*_*m*_ increases linearly with an increase in CaX_2_ content for all three glass series. An increase in glass molar volume by introducing CaF_2_ or CaCl_2_ was also found by Brauer *et al*.^31^ and Chen *et al*.^9,32^. Here, the increased slope for GPCl series is more pronounced than the equivalent GPFCl series, which is much more significant than the equivalent GPF glass series. The key factor resulting in the difference is the fact that the chloride ion is substantially larger than the fluoride ion. Therefore, the dilution of glass network structure and the expansion of glass volume by incorporating chloride are much more pronounced than by only incorporating fluoride. Meanwhile, the incorporation of mixed fluoride and chloride has an intermediate effect.

### XRD patterns of the as-quenched glasses

**Figure 5:**
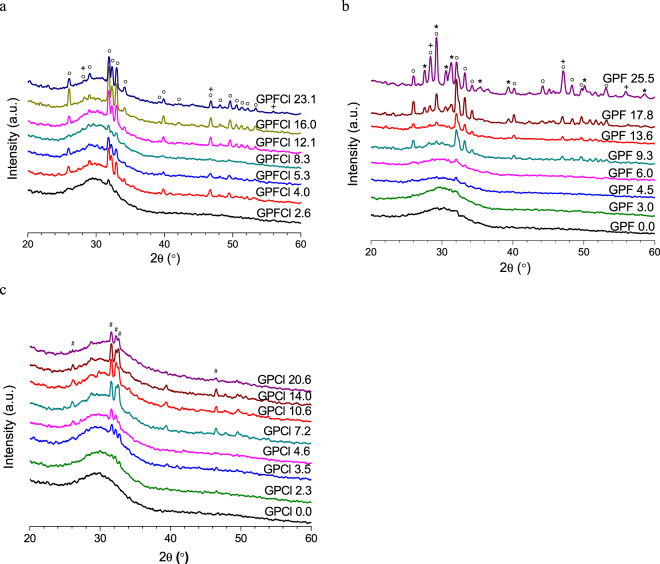
The XRD patterns of as-quenched (**a**) mixed CaF_2_ and CaCl_2_ containing glasses; (**b**) CaF_2_ containing glasses^25^; (**c**) CaCl_2_ containing glasses^23^ (^о^Ca_10_(PO_4_)_6_F_2_; *Ca_4_Si_2_O_7_F_2_; ^+^CaF_2_; ^#^Ca_10_(PO_4_)_6_(OH/Cl)_2_).

Figure 5(a) shows the XRD patterns of the as-quenched mixed CaF_2_ and CaCl_2_ containing BGs. The amorphous glass halo at 30° 2θ is found in all the CaX_2_ (X = F + Cl) containing glasses. The typical apatite diffraction lines at 25.9°, 31.8°, 32.2° and 33° 2θ (FAP: 00-034-0011) are noticed in the glasses with CaX_2_ content higher than 2.4 mol%. The additional minor diffraction lines at 28.3° and 47° 2θ possibly corresponding to fluorite (CaF_2_, 00-004-0864) are found in GPFCl16.0 and GPFCl23.1. The XRD patterns of the GPF^17^ and GPCl^16^ glass series that have been shown previously are adapted and presented in Fig. 5(b) and (c) for comparison purpose. The figures indicate that the high fluoride containing glasses show the highest crystallinity, while the mixed glasses demonstrate the highest tendency of crystallisation, i.e. the mixed glass reveal evidence for a spontaneous crystallisation at the lowest halide content compared to the other two series.

### MAS-NMR spectra of the as-quenched glasses

**Figure 6:**
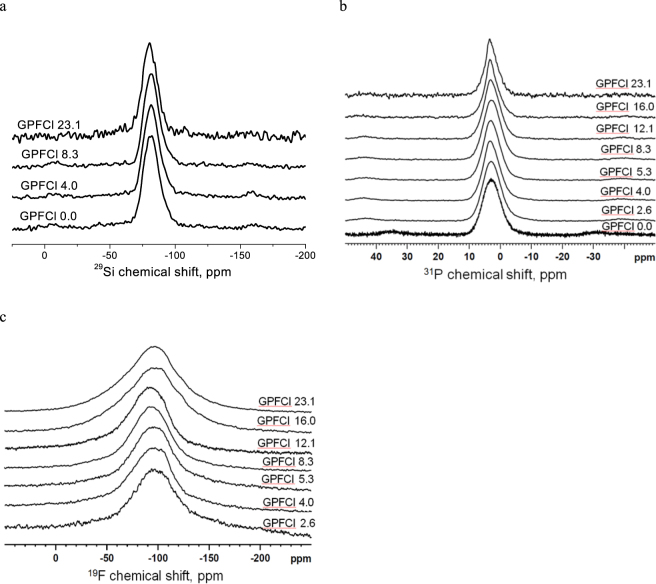
(**a**) The ^29^Si MAS-NMR spectra (**b**) ^31^P MAS-NMR spectra and (**c**) ^19^F MAS-NMR spectra of the as-quenched mixed CaF_2_ and CaCl_2_ containing glasses.

The mixed CaF_2_ and CaCl_2_ containing BGs exhibit similar ^29^Si MAS-NMR spectra, with chemical shift around −81 ppm corresponding to Q^2^ silicate units^27^ in Fig. 6(a). This suggests that all these glasses are mainly Q^2^ in structure and the incorporation of calcium halide did not cause a significant change in the glass Q silicate network structure.

The ^31^P MAS-NMR spectra of the mixed CaF_2_ and CaCl_2_ containing BGs are shown in Fig. 6(b). The single peak located in the range 3–3.3 ppm is seen in all glass spectra, suggesting that phosphate is largely present as calcium orthophosphate^27^. These peaks are slightly asymmetric, suggesting the presence of more than one signal that strongly overlap with each other. There is a change in the line shape of this signal seen with an increase in the halide content. For the glasses with CaX_2_ content higher than 2.4 mol%, the orthophosphate peak is slightly sharper and narrower with an increase in CaX_2_ content down the series. This can potentially indicate a minor presence of the crystalline phase and that the glasses with CaX_2_ content higher than 2.4 mol% tend to partially crystallise to apatite-like phase. Moreover, the peak position shifts to a slightly higher ppm value when the glasses have CaX_2_ content equal and higher than 11.3 mol%.

Figure 6(c) shows the ^19^F MAS-NMR spectra of the as-quenched mixed CaF_2_ and CaCl_2_ containing glasses. The single broad peak with a chemical shift of about −96 ppm is seen in all the glass spectra. In addition, a small shoulder at −103 ppm corresponding to FAP appears in the glass spectra with CaX_2_ contents higher than 2.4 mol%. The broad peaks are believed to contain signals from the amorphous F-Ca(n) environment in the glass, the F-Ca(3) site in the apatite structure and the overlapping spinning side bands from both signals. Note that a clear increase in the linewidth occurs in the spectra of GPFCl16.0 and GPFCl23.1. This can be attributed to the presence of an additional crystalline F-Ca(4) site at −108 ppm as a result of CaF_2_ crystallisation and its spinning side bands in the spectra.

## Discussion

The results presented above reveal that there is a remarkable similarity between all three series of bioactive glasses, in terms of their properties, resulting from the structural resemblance. This indicates that mixing two halide components in BG composition provides a powerful tool to tailor glass properties for a desired application where the beneficial presence of both fluoride and chloride is to be exploited. At the same time, there are a number of subtle dissimilarities found between the three series resulting largely from the size difference of fluoride and chloride ions and that need to be discussed.

The large chloride retention across the mixed and chloride only containing glasses is confirmed to be uniform, as seen from the Fig. 1. This large chloride retention shows a remarkable contrast to the massive chloride loss (up to 95%) during the melting of silicate glasses in the literature^24^. It is thought to be mainly contributed by the high non-bridging oxygen (NBO) fraction and the structural role of chlorine being present as Cl-Ca(n) species in these studied Q^2^ type silicate glasses^9,32^, which minimize the formation of Si-Cl bonds and inhibit the formation of SiCl_4_, subsequently prevent the loss of chlorine, while those glasses in the literature^24^ are normally highly cross-linked with a low NBO content. Generally, chlorine is also likely to be lost as metal chlorides in silicate glasses^24^, due to their low boiling point (1413 °C for NaCl, 1412 °C for MgCl_2_, 1250 °C for SrCl_2_ and 756 °C for ZnCl_2_ etc.). However, in the studied glasses, CaCl_2_ has a high boiling point (1935 °C), which is much higher than the glass firing temperatures (Table 1). This could be also beneficial to the large chloride retention.

The results show a strong linear reduction of T_g_ with addition of the halide component, regardless whether fluoride or chloride or the mixture of both is added (Fig. 2). This provides another indication that chloride losses were minimised as the T_g_ values for all three glass series with CaX_2_ (X = F/Cl/F + Cl) content less than 10 mol% are pretty much identical across the series. It is thought that the reduction in T_g_ is attributed to the formation of ‘CaX^+^’ species in the glass matrix, which behave in a similar fashion to Na^+^ cations. The formation of ‘CaX^+^’ species knocks out the ionic bridge between two NBOs by divalent calcium ions and therefore disrupts the glass network, which accounts for the observed reduction in T_g_, and facilitates glass degradation. Owing to the significant crystallisation of the high fluoride containing phases, a higher T_g_ is observed for the compositions of GPF glass series with high CaF_2_ content (>13.6 mol%). The crystallisation of fluoride containing phases effectively removes fluoride from glass matrix; therefore, the T_g_ reduction slows down in the glasses with more fluoride containing crystals.

Both the chloride retention and the nearly identical reduction in T_g_ with increasing chloride and fluoride contents are closely related to the glass structure evolution. The ^29^Si MAS-NMR spectra (Fig. 6(a)) evidently show that Q^2^ speciation of the silicate glass network in the mixed system is unaltered on addition of fluoride and chloride. Additionally, ^31^P MAS-NMR results show that Q^0^ phosphate dominates phosphorus speciation in these glasses, and little or no P-F/Cl bonds form. These results on mixed chloride/fluoride glasses structure mirror the results on the individual fluoride and chloride containing glasses reported earlier. Incorporating CaF_2_ into a SiO_2_-CaO-Na_2_O-P_2_O_5_ and SiO_2_-CaO-P_2_O_5_ or CaCl_2_ into a SiO_2_-CaO-P_2_O_5_ and SiO_2_-CaO glass system was not found to cause a change in Q^2^ speciation of bioactive silicate glasses^9,27,32,33^. Moreover, Chen *et al*.^25^ also found that phosphate existed mainly as amorphous calcium orthophosphate in the alkali free fluoride or chloride containing BGs.

The three different trends in the density values for the three different series demonstrate how fluoride and chloride can affect glass properties and how properties can be controlled via combination of the two halide components. Two opposite trends of density obtained for individual halide series were practically compensated in the mixed series resulting in only small changes within the series. The experimental density of the mixed series turned out to be within the estimated error to the density values calculated using the linear combination of the data of the individual fluoride- and chloride-only series (Figure S1, ESI).

However, the modelling of the density in these relatively simple silicate glasses remains challenging. The chloride series showed the largest deviations between the experimental density values and the values calculated based on the Doweidar model^34^ (Figure S2, ESI). The fluoride series shows a relatively good agreement between the experimental and calculated values for the high fluoride content, whereas in the mixed and chloride series the difference is higher in the compositions with high halide content. The calculations predict a stronger reduction in density for the same amount of chloride incorporated in both GPCl and GPFCl series. Although the overall increase in density with addition of calcium fluoride is consistent with the earlier observations, the Doweidar’s model did not show good agreement with the experimental density values for the fluoride-only series, which is surprising as the GPF series is simpler in terms of its atomic variety than the sodium-containing BGs studied earlier^31,35^. The Doweidar’s model is based on the binomial distribution of the silicate species, which might not necessarily be true for the one-cation system studied here. This would be interesting topic for further investigation.

T_g_ is a significant parameter, which reflects the glass structure and can indirectly predict glass solubility, degradability and hardness of glasses within certain compositional ranges^36,37^. Similarly informative is the glass molar volume; the comparison between the closely related series reported here is particularly useful. The molar volume which is used to mirror the compactness of the glass showed the potential to be used to predict glass hardness^38^. The molar volume of the glasses increases on adding a halide component, which is in a good agreement with the reduction in T_g_ with an increase in CaX_2_ content. However, the rate of this increase is distinctly different; chloride is shown to be most efficient in expansion of the glass volume compared to a mixture with fluoride and chloride, and fluoride alone, which only causes small increase in glass molar volume.

The molar volume values of the mixed series estimated from the data on individual series of fluoride and chloride only fall within the 3% error on the values obtained from the experimental data on the mixed series. Thus, on incorporation of CaX_2_, the glass hardness would be expected to decrease, as a consequence of a reduced compactness of the glass by expanding the glass volume, and the rate of this decrease can be controlled via ratio between CaF_2_ and CaCl_2_ added to the glass. Moreover, the glasses with larger molar volume are expected to have a faster glass degradation rate and a lower crystallinity.

Incorporating a bigger chloride ion as opposed to fluoride is expected to result in a reduced tendency to crystallisation, since the lattice energies of the equivalent crystalline phase is likely to be larger, e.g. ClAP *vs* FAP^39^, and a large chloride ion is less likely to order calcium cations around itself than a smaller fluoride ion. Consequently, larger amounts of CaCl_2_ than CaF_2_ can be incorporated into the glasses without significant crystallisation occurring during quenching. In this work, it was expected that the GPF series would crystallise most readily, while the GPCl series crystallises least readily and the mixed GPFCl series will have an intermediate crystallisation tendency.

The XRD patterns of the GPF glass series do show a high crystallisation tendency; FAP, cuspidine and fluorite crystallise in sequence when the CaF_2_ content ≥ 9.3 mol%^17^. Unlike GPF glass series, on incorporating CaCl_2_, the tendency of the glasses to crystallise is suppressed; all the glasses (up to 16.7 mol% CaCl_2_) from GPCl series are largely amorphous. The minor crystalline phase of the mixed hydroxy-chlorapatite detected by XRD in chloride series is thought to form by reaction with atmospheric water on the surface of samples during the course of acquiring the XRD patterns^25^.

In the case of GPFCl series, instead of seeing an expected intermediate crystallisation tendency in between GPF and GPCl glass series, a stronger crystallisation tendency is evidenced from XRD and NMR compared to the equivalent GPF glass series. Spontaneous crystallisation is detected in composition with the CaX_2_ content as little as 2.4 mol% and above. This is likely due to the fact that the presence of chloride expands the glass volume effectively and therefore to some extent facilitates the arrangement of calcium cations around a fluoride ion. The crystalline phase identified in the mixed glasses is FAP with addition of CaF_2_ phase in GPFCl16.0 and GPFCl23.1 glasses. The amount of the fluoride containing crystalline phase remains minor fraction for all the compositions, as seen from the XRD patterns (Fig. 5) and NMR spectra (Fig. 6(b) and (c)). Therefore, the GPFCl glasses are expected to be highly bioactive by combining the benefits from both fluoride and chloride. The presence of chloride expands glass structure and promotes rapid glass degradation upon immersion, while fluoride stimulates FAP formation.

## Conclusion

It is clear that the properties of mixed CaF_2_ and CaCl_2_ containing BGs are contributed from the presence of both CaF_2_ and CaCl_2_. T_g_ decreases with an increase in CaX_2_ content. In contrast, the glass molar volume increases significantly with increasing CaX_2_ content, suggesting that both fluoride and chloride expand the glass volume and dilute glass network, therefore facilitate glass degradation. However, the expansion effect by fluoride is much smaller than the equivalent chloride as the fluoride ion is substantially smaller than the chloride ion. The significant expansion of glass volume associated with the addition of chloride leads to a decrease in the glass crystallinity but an increase in the tendency of crystallisation. With the exception of glass GPFCl2.6, which is largely amorphous, the studied glasses are partially crystallised to fluorapatite during quenching. In addition, CaF_2_ is also found in glass GPFCl16.0 and 23.1. The nearly constant chemical shift of the ^29^Si MAS-NMR spectra at -80ppm suggests that the presence of CaX_2_ causes no significant change in the glass silicate network structure, which is comprised of mainly Q^2^ species. The fluoride and the chloride are present as F-Ca(n) and Cl-Ca(n) species in these glasses and no detectable Si-F or Si-Cl species were found. The ^31^P MAS-NMR spectra indicate that phosphate is dominantly present as orthophosphate in the glasses. Based on these results it is possible to design oxyhalide containing silicate glass and tailor their properties for different dental and medical applications.

## Electronic supplementary material

Supplementary Information
