# Supplementary material for: New Insight into Mixing Fluoride and Chloride in Bioactive Silicate Glasses
Source: Sci Rep. 2018 Jan 22;8:1316. doi: 10.1038/s41598-018-19544-2 (PMC5778077; doi:10.1038/s41598-018-19544-2)
Supplement: Supplementary file 1 — Supplementary Information [file 41598_2018_19544_MOESM1_ESM.doc]

New Insight into Mixing Fluoride and Chloride in Bioactive Silicate Glasses

Xiaojing Chen^1,2^, Xiaohui Chen^3^, Alfonso Pedone^4^, David Apperley^5^, Robert G. Hill^2^, Natalia Karpukhina^2^*

^1^Xiangya Stomatological Hospital & School of Stomatology, Central South University, Changsha, Hunan 410078, P.R. China

^2^Dental Physical Sciences, Institute of Dentistry, Queen Mary University of London, Mile End Road, London E1 4NS, United Kingdom

^3^Division of Dentistry, School of Medical Sciences, University of Manchester, Manchester M13 9PL, United Kingdom

^4^Dipartimento di Scienze Chimiche e Geologiche, Università di Modena e Reggio Emilia, Via G. Campi 103, 41125 Modena, Italy

^5^Department of Chemistry, Durham University, South Road, Durham, DH1 3LE, United Kingdom

*Correspondence to [n.karpukhina@qmul.ac.uk](mailto:n.karpukhina@qmul.ac.uk) (Natalia Karpukhina)

Table S1. The as-designed glass compositions (in Mol%). ^1,2^

| **Glass** | **SiO_2_** | **CaO** | **P_2_O_5_** | **CaF_2_** | **CaCl_2_** | **T_firing_ (°C)** | **NC** |
| --- | --- | --- | --- | --- | --- | --- | --- |
| GPF/GPCl 0.0 | 38.1 | 55.5 | 6.3 | - | - | 1550 | 2.08 |
| GPF 3.0 | 37.0 | 53.9 | 6.1 | 3.0 | - | 1550 |
| GPF 4.5 | 36.4 | 53.0 | 6.0 | 4.5 | - | 1500 |
| GPF 6.0 | 35.9 | 52.2 | 6.0 | 6.0 | - | 1500 |
| GPF 9.3 | 34.6 | 50.4 | 5.7 | 9.3 | - | 1500 |
| GPF 13.6 | 32.9 | 48.0 | 5.5 | 13.6 | - | 1500 |
| GPF 17.8 | 31.4 | 45.7 | 5.2 | 17.8 | - | 1500 |
| GPF 25.5 | 28.4 | 41.4 | 4.7 | 25.5 | - | 1500 |
| GPCl 2.3 | 37.3 | 54.3 | 6.2 | - | 2.3 | 1500 |
| GPCl 3.5 | 36.8 | 53.6 | 6.1 | - | 3.5 | 1500 |
| GPCl 4.6 | 36.4 | 53.0 | 6.0 | - | 4.6 | 1500 |
| GPCl 7.2 | 35.4 | 51.6 | 5.9 | - | 7.2 | 1480 |
| GPCl 10.6 | 34.1 | 49.6 | 5.7 | - | 10.6 | 1480 |
| GPCl 14.0 | 32.8 | 47.7 | 5.4 | - | 14.0 | 1480 |
| GPCl 20.6 | 30.3 | 44.1 | 5.0 | - | 20.6 | 1450 |

Figure S1. The comparison between the experimental density (D_e_) and the calculated density (D_c_) based on the density values of individual series assuming an equal effect of fluoride and chloride on density for mixed fluoride and chloride containing glasses.

Figure S2. The experimental density (D_e_, bigger symbols) and the calculated density (D_c_, smaller symbols) based on Doweidar for all three glass series profiled as a function of the actual CaX_2_ (X=F/Cl/F+Cl) content. The solid lines correspond to the liner relationships between calculated density and actual CaX_2_ content.

Figure S3. The FTIR spectra of as-quenched CaX_2_ (X=F+Cl) containing glasses.
